# Observation of Magnetic Domains in Amorphous Magnetic Wires with a Diameter of 10 μm Used in GSR Sensors

**DOI:** 10.3390/s23073506

**Published:** 2023-03-27

**Authors:** Masaaki Takezawa, Yuki Harada, Yoshinobu Honkura, Shinpei Honkura

**Affiliations:** 1 Kyushu Institute of Technology, Fukuoka 804-0015, Japan; 2 Magnedesign Corporation, Nagoya 466-0059, Japan

**Keywords:** magnetic domains, amorphous wire, magnetic sensor, GSR sensor

## Abstract

The core of a Gigahertz Spin Rotation (GSR) sensor, a compact and highly sensitive magnetic sensor, is composed of Co–Fe-based amorphous magnetic wire with a diameter of 10 μm. Observations of the magnetic domain structure showed that this magnetic wire has unusual magnetic noise characteristics. Bamboo-shaped magnetic domains a few hundred micrometers in width were observed to form inside the wire, and smaller domains a few micrometers across were observed to form inside these larger domains. The magnetic domain pattern changed abruptly when an external magnetic field was applied to the wire. Herein is shown how these changes may be a source of magnetic noise in the wire.

## 1. Introduction

Magnetic sensors are used in various automotive, medical, and data-storage applications, and their performance is expected to be improved. The magnetic core of a Gigahertz Spin Rotation (GSR) sensor is composed of amorphous magnetic wire, on which a detection coil is wound [1,2,3]. Amorphous magnetic wires are known to have a characteristic magnetic domain structure and have been extensively studied [4,5,6,7,8,9,10,11,12,13,14,15,16,17,18,19]. Takajo and Yamasaki et al. reported that Fe-based wires have maze-like magnetic domains near the surface and magnetic domains parallel to the axis in the core [11,15,17,18]. It is also known that the magnetic domain structure of Co–Fe-based wires changes with the Co content, with circumferential bamboo-shaped magnetic domain structures occurring near the surface and triangular closed domains being observed on the polished surface [17]. Such disturbances in the magnetic domain structure, as well as local vortex-like structures, can be a source of noise in GSR sensors that use this type of wire. Thermal noise and the magnetostriction of magnetic materials have been reported to be one of the causes of noise in GMI sensors and fluxgate magnetic sensors using amorphous magnetic materials [20,21,22,23,24,25,26,27]. However, although observations of the magnetic domains of wires with diameters of 50 to 100 µm have been made, there have been few studies of the domains of amorphous magnetic wires with diameters of less than 50 µm, such as those used in GSR sensors [4,5,6]. High-resolution magnetic domain observation techniques using Magnetic Force Microscope (MFM) and Transmission Electron Microscope (TEM) [6,28] are necessary to observe magnetic domains in thin wires. On the other hand, the magnetic domain observation technique using the Kerr effect has the advantage of the in situ observation of magnetic domain structure changes due to applying a magnetic field. However, it is inferior to MFM and TEM regarding spatial resolution. The authors improved the high-spatial-resolution magnetic domain observation technique using the Kerr effect by using ultraviolet light and image processing [29,30]. In this study, we aimed to clarify the source of noise in GSR sensors by making observations of the magnetic domain structure of a 10 µm diameter Co–Fe-based magnetic amorphous wire.

## 2. Experimental Procedure

The wire used for our observations was an as-cast Co–Fe–Si–B amorphous magnetic wire that had a diameter of 10 µm. A vibrating sample magnetometer was used to measure hysteresis loops, and a Kerr effect microscope was used to observe the magnetic domains. To polish the sample to have a mirror-like surface, sections of wire approximately 5 mm long were embedded in epoxy resin, mechanically polished using a diamond paste, then chemically polished using alumina and colloidal silica. An external magnetic field was applied using a Helmholtz coil; the changes that then occurred in the magnetic domain structure were observed. The magnetic domain contrast was enhanced by differential processing of magnetic domain images [31].

## 3. Results and Discussion

### 3.1. Hysteresis Loop

**Figure 1:**
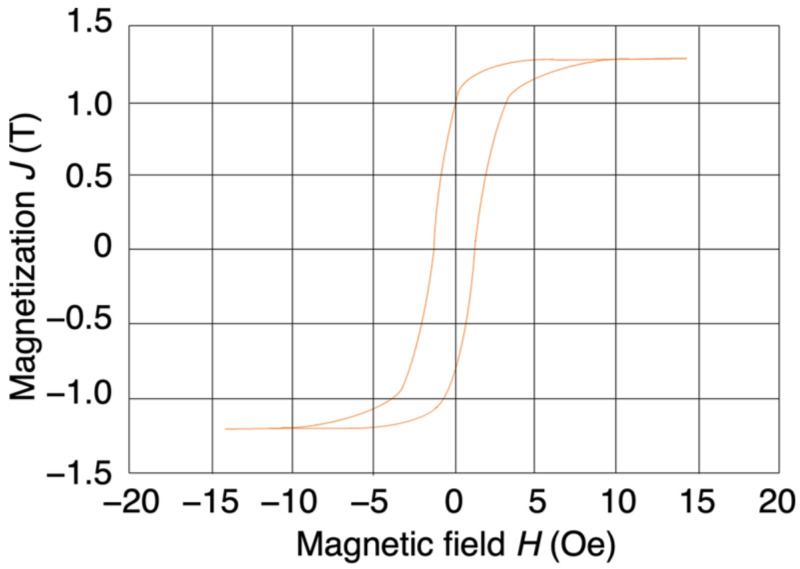
Hysteresis loop of a Co–Fe–Si–B amorphous magnetic wire with a diameter of 10 µm.

The measured hysteresis loop of the Co–Fe–Si–B amorphous magnetic wire is shown in Figure 1. The measurement direction was along the wire’s axial direction, and the coercive force was about 0.86 Oe. It can be seen that the saturation magnetization was about 1.3 T, and that this could be reached by applying an external magnetic field of about 10 Oe. These results indicate that the wire had excellent soft magnetic properties.

### 3.2. Magnetic Domains

#### 3.2.1. Macroscopic Observations of Magnetic Domains

**Figure 2:**
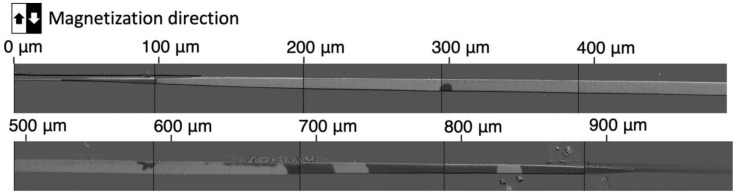
Overall view of the polished surface of an amorphous magnetic wire.

Figure 2 shows the magnetic domains that were observed on the polished surface of the magnetic wire. The center of the 5 mm long wire was polished over a length of about 1 mm. The width of the polished surface is narrower at the left- and right-hand ends of the polished area, indicating that the polished depth is smaller at the ends and greater in the center. The bright and dark magnetic domains correspond to upward and downward magnetization, respectively, indicating that the orientation of the magnetization is transverse to the axis of the wire. The domain configuration also indicates that the magnetization is oriented in this direction at the polished surface, i.e., the magnetic domains can be considered to form a bamboo-like structure [9,11,12,15,17] with a non-uniform width.

**Figure 3:**
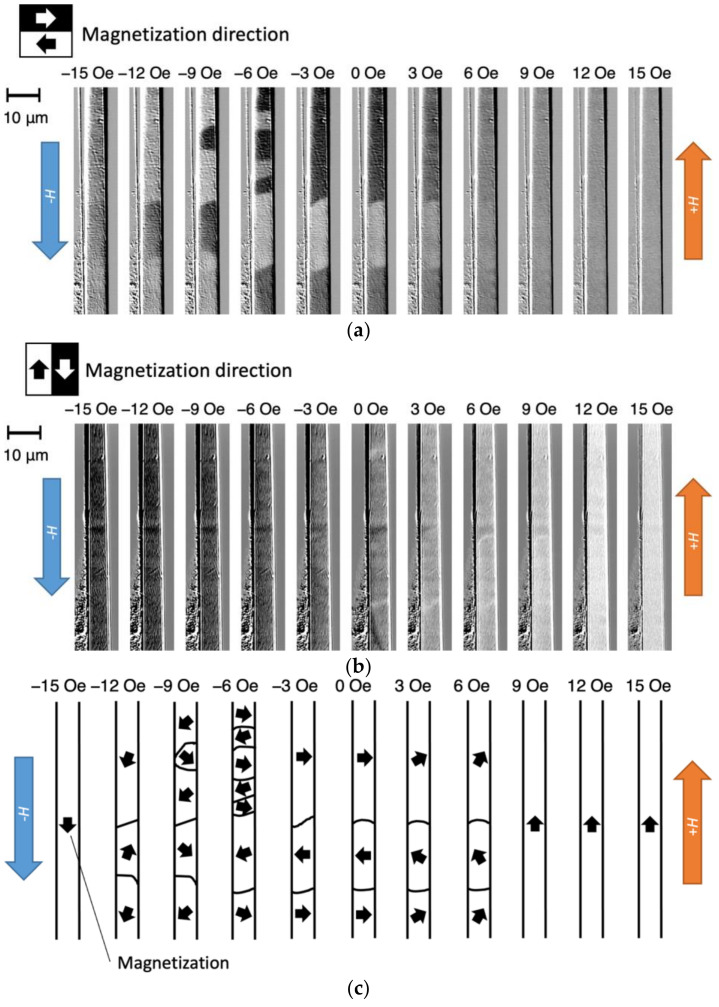
Application of an external magnetic field in the axial direction around 700 to 800 μm from the left-hand end of the wire shown in Figure 2: (**a**) radial magnetic domain pattern; (**b**) axial magnetic domain pattern; (**c**) and inferred magnetization direction.

Figure 3 shows the change in the magnetic domain that occurred around 700 to 800 μm from the left-hand end of the wire when the magnetic field, *H*, that was applied in the axial direction was varied from +15 to −15 Oe. The bright and dark areas in Figure 3a correspond to the leftward and rightward magnetization components, respectively. In the remanent magnetization state (*H* = 0), the magnetization direction alternates between left and right in the radial direction of the wire. When *H* reaches either −15 or +15 Oe, the original bamboo-like domain structure, which is characteristic of wires containing Co [9,17], disappears, and the direction of the domain becomes uniform throughout the wire. This indicates that the application of a magnetic field causes rotation of the magnetization in the wire’s axial direction, resulting in magnetic saturation.

Figure 3b shows the same field of view as Figure 3a; in this case, the bright and dark areas correspond to the detected magnetization component in the vertical direction in the figure. Since a line-shaped contrast can be observed at the boundary of the magnetic domain in Figure 3a, it is considered that the magnetization forms a Néel magnetic wall that rotates in the plane of the polished surface in the vicinity of the magnetic wall.

Based on these results, a model showing the structure of the magnetic domains in the wire was constructed and is shown in Figure 3c. The direction of the magnetization is along the wire axis, and magnetic saturation occurs at *H* = +15 Oe. When the magnetic field is reduced to +6 Oe, the magnetization rotates so that it is directed to the left and right of the wire radius and magnetic domain walls appear, thus forming a striped magnetic domain structure with the magnetization facing the wire radius in the remanent magnetization state. When the external magnetic field is subsequently increased in the negative direction, new magnetic domains are nucleated; in addition, continuous rotation of the magnetization and movement of the magnetic domain walls occurs. Thus, a discontinuous magnetic domain change occurs inside the wire during the magnetization process in addition to the rotation of the magnetization and magnetic wall movement. These sudden changes in the magnetic domain structure can produce noise in magnetic sensors. We consider that the nucleation of magnetic domains occurs when a magnetic field is applied in the axial direction of the wire because the easy axis of magnetization is inclined from the radial direction of the wire.

#### 3.2.2. Microscopic Observations of Magnetic Domains

**Figure 4:**
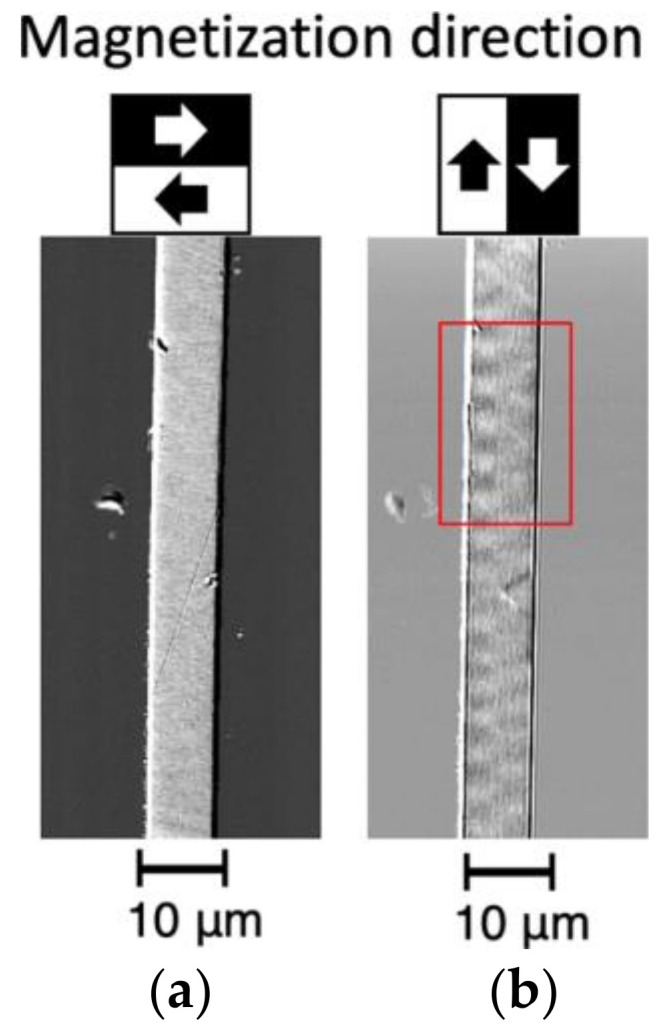
Image of magnetic domains around 400 to 500 μm from the left-hand end of the wire shown in Figure 2: (**a**) radial magnetic domain pattern and (**b**) axial magnetic domain pattern.

Figure 4 is an image of the magnetic domains around 400 to 500 μm from the left-hand end of the wire shown in Figure 2. The surface was polished to a greater depth than in the case of the wire shown in Figure 3. Figure 4a shows the magnetic domain contrast in the radial direction; it can be seen that there is uniform leftward magnetization. Figure 4b shows the magnetic domain contrast in the axial direction of the wire; a complex striped pattern of several µm in size can be observed. It can be seen that an even finer magnetic domain structure with domains a few micrometers across formed inside the bamboo-like domain structures described in Section 3.2.1 (which had widths of several hundred micrometers). The magnetization in these smaller domains is directed along the radius of the wire; however, Figure 4b shows that it has a small axial component.

**Figure 5:**
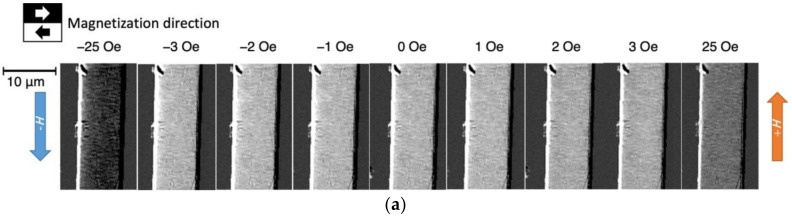

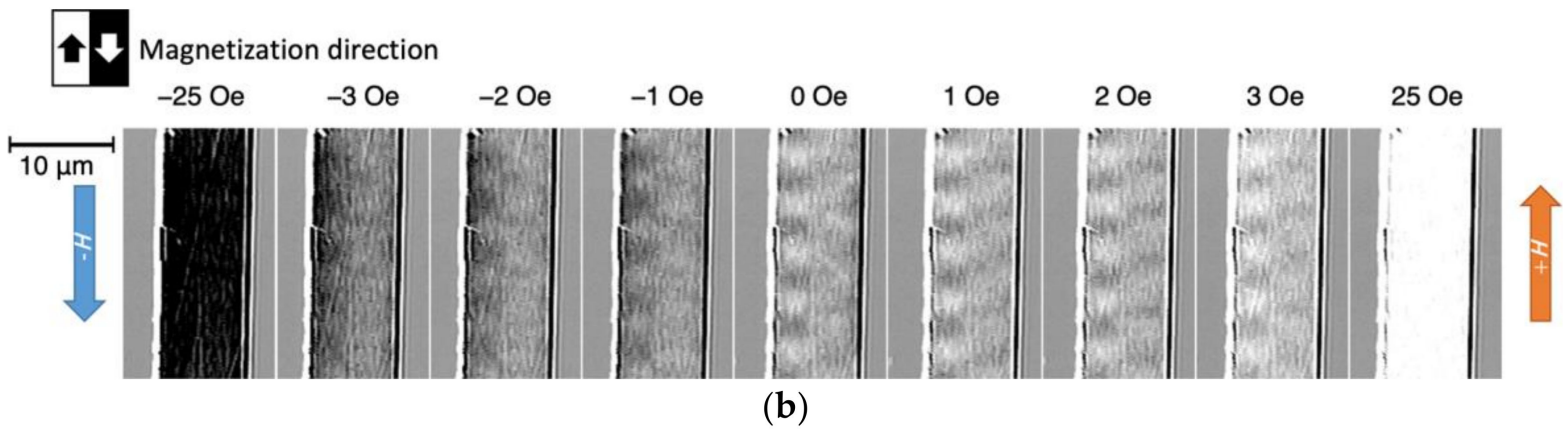
Application of an external magnetic field in the axial direction: (**a**) radial magnetic domain pattern and (**b**) axial magnetic domain pattern.

To investigate the magnetization process for this fine magnetic domain structure, we observed the change in the magnetic domain structure when a magnetic field directed along the wire axis was applied. Figure 5 shows the change in the magnetic domain pattern within the area enclosed by the red box in Figure 4b when the magnetic field in the direction of the wire axis was varied from +25 to −25 Oe. Figure 5a,b show the magnetic domain pattern in the radial and axial directions of the wire, respectively. 

Figure 5a shows no difference in the magnetization direction in the radial direction throughout the field of view. At around *H* = 0 Oe, the direction of the magnetization is to the left.

In contrast, Figure 5b shows that fine magnetic domains a few micrometers across with magnetization components in the axial direction of the wire were observed near *H* = 0 Oe. The magnetic domain contrast becomes weaker as the magnetic field increases and disappears when magnetic saturation is reached. In addition, when the magnetic field direction changes from positive to negative, the magnetic domain pattern reverses; in other words, when the magnetic field changes from 0 to −1 Oe, a sharp reversal of the magnetization component in the direction of the wire axis occurs. This abrupt change in the magnetic domain pattern is a possible source of noise in the GSR sensor element.

**Figure 6:**
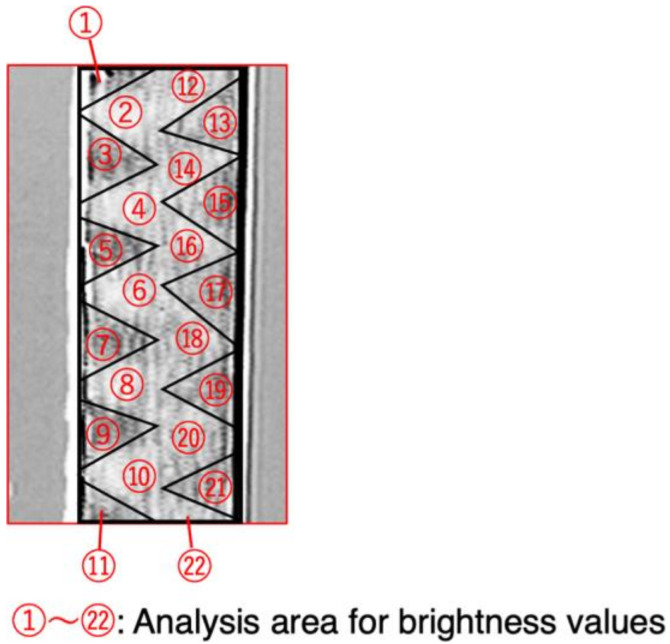
Section of wire shown in Figure 5a divided into 22 regions according to the magnetic domain pattern.

Figure 6 shows the remanent magnetization state for the wire shown in Figure 5a divided into 22 regions according to the magnetic domain pattern. The brightness values of these 22 regions were used to derive the orientation of the magnetization. The brightness values were expressed as numbers in the range 0 to 255 depending on the wire axis component of magnetization, with a higher value indicating a brighter region. For the situation shown in Figure 5a, the overall brightness of the wire when a magnetic field of +25 Oe was applied was 124.6; for a field of −25 Oe, it was 44.5. These states were defined as corresponding to angles of +90° and −90° relative to the wire radius direction (left direction), respectively. Using these definitions, an angle of 0° had a brightness of 84.5. The formula used to derive the amount of rotation, *θ*, was

(1) $$\theta =\sin^{-1}\left\{\frac{\left(\mathrm{Brightness}\;\mathrm{value}\right)-84.5}{124.6-84.5}\right\}$$

**Figure 7:**
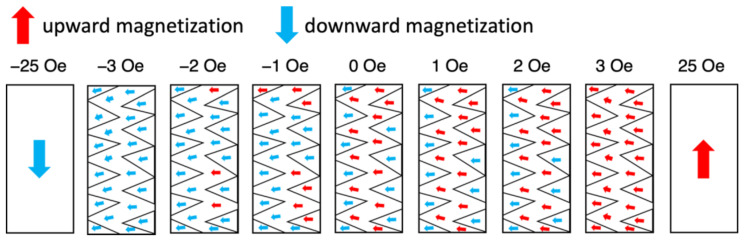
Magnetization directions calculated from the brightness values for the section of wire shown in Figure 5a.

Figure 7 shows the magnetization directions that were calculated from the measured brightness values. The red and blue arrows indicate upward and downward magnetization, respectively. In the remanent magnetization state, the magnetization is oriented to the left throughout the section of wire. However, there is an upward component inside the zigzag-shaped magnetic domain in the center of the wire and a downward component inside the triangular magnetic domains at the edges. When the magnetic field changes from 0 to −1 Oe, most of the regions of upward magnetization change to regions of downward magnetization. 

**Figure 8:**
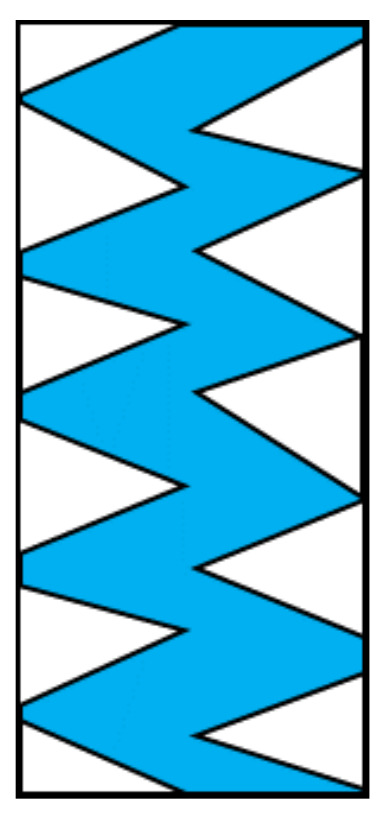
Diagram showing areas with an abrupt change in the direction of magnetization.

The region where this rapid change in the magnetization direction occurs is shown in blue in Figure 8. In can be seen that a sharp change in the direction of the magnetization in the direction of the wire axis occurred in the zigzag-shaped region in the center. This region occupies about half the area of the entire wire, and this abrupt change in the magnetic domain structure will generate a large output voltage at the detection coil that is wound around the wire in the sensor element, thus causing an increase in noise.

## 4. Conclusions

Observations were made of the magnetic domain structure on the polished surface of a Co–Fe-based amorphous magnetic wire that had a diameter of 10 µm. Bamboo-shaped magnetic domains a few hundred micrometers in width formed inside the wire. This macroscopic domain structure causes rotation of the magnetization and a magnetic wall shift during reversal of the magnetization; in addition, the nucleation of multiple magnetic domains causes discontinuous and abrupt magnetization changes. These changes in the domain structure can produce noise in magnetic sensors. Inside the bamboo-shaped magnetic domains, there is an additional, finer domain structure consisting of domains a few micrometers across. Within the micrometer-scale zigzag-shaped region observed in the center of the wire, large changes in the direction of the magnetization occur. Such a fine magnetic domain structure inside the bamboo-shaped magnetic domain was not observed in the 100 µm diameter magnetic wires observed in the past. These changes in direction and the magnetic domain structure are another source of noise in magnetic sensors.

In this study, observations of magnetic domains were performed at the polished surface of an amorphous magnetic wire. Further investigations, such as three-dimensional magnetic domain observations [32,33,34,35,36], are needed to clarify the effect of polishing on the magnetic domain structure. In the future, it would be desirable to experimentally measure the noise of the GSR sensor and compare it directly with the changes of the magnetic domain structure. Nevertheless, we clarified one of the causes of noise in GSR sensors by observing the magnetic domains in microwires. In subsequent studies, we plan to study the effect of heat treatment on the magnetic domain structure of amorphous magnetic wires and GSR sensor noise.

## Data Availability

All data generated or analyzed during this study are included in this article.
